# Hermite Functions, Lie Groups and Fourier Analysis

**DOI:** 10.3390/e20110816

**Published:** 2018-10-23

**Authors:** Enrico Celeghini, Manuel Gadella, Mariano A. del Olmo

**Affiliations:** 1 Dipartimento di Fisica, Università di Firenze and INFN-Sezione di Firenze, 50019 Sesto Fiorentino, Firenze, Italy; 2 Departamento de Física Teórica, Atómica y Optica, Universidad de Valladolid, 47011 Valladolid, Spain; 3 Instituto de Matemáticas, Universidad de Valladolid (IMUVA), 47011 Valladolid, Spain

**Keywords:** Fourier analysis, special functions, rigged Hilbert spaces, quantum mechanics, signal processing

## Abstract

In this paper, we present recent results in harmonic analysis in the real line R and in the half-line $R^{+}$, which show a closed relation between Hermite and Laguerre functions, respectively, their symmetry groups and Fourier analysis. This can be done in terms of a unified framework based on the use of rigged Hilbert spaces. We find a relation between the universal enveloping algebra of the symmetry groups with the fractional Fourier transform. The results obtained are relevant in quantum mechanics as well as in signal processing as Fourier analysis has a close relation with signal filters. In addition, we introduce some new results concerning a discretized Fourier transform on the circle. We introduce new functions on the circle constructed with the use of Hermite functions with interesting properties under Fourier transformations.

## 1. Introduction

The seminal work by Fourier of 1807, published in 1822 [1], about the solution of the heat equation had a deep impact in physics and mathematics as is well known. Roughly speaking, the Fourier method decomposes functions into a superposition of “special functions” [2,3]. In particular, trigonometric functions were used by Fourier himself for this purpose. In addition, the Fourier method makes use of other types of special functions; each of these types is often related with a group. Then, these special functions have symmetry properties, which are inherited from the corresponding group. For instance, this is the way in which harmonic analysis appears in group representation theory [4]. An interesting aspect of Fourier analysis is the decomposition of Hilbert space vectors, quite often represented by square integrable functions on some domain, into an orthogonal basis. This generalizes both the standard Fourier analysis of trigonometric series and the decomposition of a vector in terms of an algebraic basis of linearly independent vectors. Another generalization is the decomposition of a self-adjoint or normal operator on a Hilbert space in terms of spectral measures, say through the spectral representation theorem. We are mainly interested in these generalizations concerning Hilbert space vectors and operators.

In recent works [5,6], we started an attempt to reformulate the harmonic analysis on the real line to obtain a global description of the Hermite functions, the Weyl–Heisenberg Lie algebra and the Fourier analysis in the framework of rigged Hilbert spaces (RHS) that we present here in a more formal way. As is well known, the Fourier transform relates two continuous bases which are used in the description of one-dimensional quantum systems on the whole real line. These are the coordinate and momentum representations, naturally connected with the position and the momentum operator [7,8], respectively. They span the Weyl–Heisenberg algebra together with the identity operator. Moreover, these two continuous bases can be related with a discrete orthonormal basis labeled by the natural numbers via the Hermite functions. In consequence, we have continuous and discrete bases within the same framework. However, only discrete bases as complete orthonormal sets have a precise meaning in Hilbert space. If we have a structure allowing to work with these types of bases and to find relations among them, one needs to extend the Hilbert space to a more general structure called the rigged Hilbert space.

The fundamental message of the present paper is to show how a class of different and apparently unrelated mathematical objects, such as classical orthogonal polynomials, Lie algebras, Fourier analysis, continuous and discrete bases and RHS, can be fully wived as a branch of harmonic analysis, with applications in quantum mechanics and signal processing, among other possible applications.

We have mentioned that the mathematical concept of RHS is very important in our work. It has been introduced by Gelfand and collaborators [9] proving (although Maurin [10]) the nuclear spectral theorem as was heuristically introduced by Dirac [11]. It is also generally accepted that the eigenfunction expansions and the Dirac formalism are generalizations of the Fourier analysis for which we need RHS [10,12]. It is also known that the spectral theory of infinitesimal operators of an arbitrary unitary representation of a Lie group also need RHS [10,12]. In the physics literature, the similarities between the Dirac formalism, classical Fourier analysis and generalized Fourier transforms have been discussed within the RHS framework [13,14]. Another application of RHS, which has a particular importance in our presentation, is signal processing. In particular, in the electrical engineering literature, these aspects have been discussed in [15,16,17,18].

Since the average physicists may not be acquainted with the concept of RHS, let us give a definition and some remarks on this concept. A rigged Hilbert space or Gelfand triple is a set of three vector spaces

$$\Phi \subset H\subset \Phi^{\times },$$

where H is an infinite dimensional separable Hilbert space, Φ is a topological vector space endowed with a topology finer than the Hilbert space topology and dense on H with the Hilbert space topology, and $\Phi^{\times }$ is the dual of Φ (i.e., the space of linear (or antilinear) continuous mappings from Φ into the complex numbers C) and it is endowed with a topology compatible with the pair $\left(\Phi ,\Phi^{\times }\right)$.

The formulation of quantum mechanics in terms of RHS was introduced by Bohm and Roberts in the sixties of the last century and further developed later [19,20,21,22,23,24,25,26]. Continuous bases are not well defined in Φ and H but only in $\Phi^{\times }$. The action of a functional $F\in \Phi^{\times }$ on a vector φ∈Φ is written as 〈φ|F〉 for keeping up with the Dirac notation. Since we will consider the scalar product on Hilbert space antilinear to the left, we shall assume the antilinearity of the elements in $\Phi^{\times }$.

The first part of the present paper is devoted to a review of a previous work by the authors [6] concerning to the above-mentioned extension of Fourier analysis on the real line with the use of special functions such us Hermite functions, which will be here for our main example. This is studied in Section 2. The use of the Fractional Fourier transform (FFT) in this analysis is discussed in Section 3.

In addition, we give a second example in which the real line has been replaced by the semi-axis $R^{+}\equiv \left[0,\infty \right)$ and Hermite functions by Laguerre functions. In this latter case, we construct two different Fourier-like transforms $T^{\pm }$ and their eigenvectors are functions on the positive half-line. This is given in Section 4 and Section 5. Extensions to $R^{n}$ using or not spherical coordinates are also possible, although we shall not consider this option in the present manuscript [27]. In Reference [28], we revisited the harmonic analysis on the group SO(2) using RHS. Furthermore, in Reference [29], we introduce a new realization of the group SU(2) in the plane in terms of the associated Laguerre polynomials.

In Section 4, we introduce some new results concerning harmonic analysis on the circle. We construct new functions on the circle using Hermite functions and taking advantage of their properties. Again, these new functions give a unitary view of different mathematical objects that are often considered as unrelated: Fourier transform, discrete Fourier transform, Hermite functions and RHS.

To understand the importance of the present research, let us remark that Hermite and Laguerre functions are bases of spaces of square integrable functions, no matter whether real or complex, defined on R and $R^{+}$, respectively. Square integrable real and complex (wave) functions play a similar role in signal processing and quantum mechanics, respectively. In addition, the interest of signal processing comes after the definition of two new types of filters. One is based in restrictions to subspaces of $L^{2}\left(R\right)$ or $L^{2}\left(R^{+}\right)$. We have systematically constructed these filters by the use of the FFT. The other requires choosing low values of the index *n* in the span of a given function by either Hermite or Laguerre functions (we may also use a combination thereof). These filters remove noise or other spurious effects from the signal or the wave function.

In addition, since the basic operators related with these functions span some Lie algebras, such as the io(2) [30] for the Hermite functions and the su(1,1) for the Laguerre functions, we can introduce a richer space of operators on $L^{2}\left(R\right)$ or $L^{2}\left(R^{+}\right)$, related to the universal enveloping algebra (UEA) of io(2) or su(1,1), respectively [31].

These operator spaces connect functions describing the time evolution of the states under filters or some kind of interaction.

Finally, we would like to add that this discussion may be related with some integral transforms of the type Fourier-like, Laplace-like or Sumudu-like transforms [32,33,34,35,36].

## 2. Harmonic Analysis on R

The first example of Fourier analysis and its relation with group theory is provided by the translation group in one spatial dimension $T_{1}≃R$ (for the group SO(2) see [28]). The action of its unitary irreducible representations, R, on the continuous basis ${\{|p\rangle \}}_{p\in R}$, given by the eigenvectors of the infinitesimal generator *P* of the group, is given by

(1) $$R\left(x\right)|p\rangle =e^{-iPx}\;|p\rangle =e^{-ipx}\;|p\rangle \;;\;P\;|p\rangle =p\;|p\rangle ,\;\forall p\in R,\forall x\in T_{1}.$$

The vectors of the basis {|p〉} verify

(2) $$\langle p|p^{\prime }\rangle =\sqrt{2\pi }\;\delta \left(p-p^{\prime }\right),\;\frac{1}{\sqrt{2\pi }}\int_{-\infty }^{\infty }|p\rangle \langle p|\;dp=I.$$

Considering the position operator *X* and a continuous basis ${\{|x\rangle \}}_{x\in R}$ of its eigenvectors, i.e.,

(3) $$X\;|x\rangle =x\;|x\rangle ,\;\forall x\in R≃T_{1}.$$

Via the Fourier transform, we can relate both (conjugate) bases {|p〉} and {|x〉}

(4) $$|x\rangle =\frac{1}{\sqrt{2\pi }}\int_{-\infty }^{\infty }\;e^{-ipx}\;|p\rangle \;dp,\;|p\rangle =\frac{1}{\sqrt{2\pi }}\int_{-\infty }^{\infty }\;e^{ipx}\;|x\rangle \;dx,$$

such that we find for the basis {|x〉} that

(5) $$\langle x|x^{\prime }\rangle =\sqrt{2\pi }\;\delta \left(x-x^{\prime }\right),\;\frac{1}{\sqrt{2\pi }}\int_{-\infty }^{\infty }|x\rangle \langle x|\;dx=I.$$

Moreover, *X*, *P* together with I determine the Weyl–Heisenberg algebra

(6) [X,P]=iI,[·,I]=0.

For more details, see Reference [37].

### 2.1. Hermite Functions and the Group IO(2)

Now, we consider the inhomogeneous orthogonal group IO(2) which is isomorphic to the Euclidean group in the plane, E(2). In the study of the ray representations [38,39,40], we have to deal with the central extended group [30]. Here, we use a non-standard technique related to the projective representations of IO(2) by considering the algebra of the harmonic oscillator that it is isomorphic to the central extension mentioned above. To proceed, let us consider the operators

$$a:=\frac{1}{\sqrt{2}}\;\left(X+iP\right),\;a^{+}:=\frac{1}{\sqrt{2}}\;\left(X-iP\right),\;N:=a\;a^{+},\;I,$$

which determine the Lie commutators

$$\left[a,a^{+}\right]=I,\;\left[N,a\right]=-a,\;\left[N,a^{+}\right]=a^{+},\;\left[I,\cdot \right]=0,$$

and the quadratic Casimir

$$C=\left\{a,a^{+}\right\}-2\left(N+1/2\right)\;I.$$

In the representation with C=0, we obtain the differential equation

(7) $$C\;K_{n}\left(x\right)\equiv \left(-D_{x}^{2}+X^{2}-\left(2N+1\right)\right)\;K_{n}\left(x\right)=0,$$

where $P=-i\;D_{X}=-i\;d/dx$ and *N* is a kind of number operator such that for each index n∈N, $N\;K_{n}\left(x\right)=n\;K_{n}\left(x\right)$, where $K_{n}\left(x\right)$ are solutions of the differential Equation (7). These solutions are the Hermite functions

(8) $$K_{n}\left(x\right):=\frac{e^{-x^{2}/2}}{\sqrt{2^{n}n!\sqrt{\pi }}}\;H_{n}\left(x\right),$$

with $H_{n}\left(x\right)$ the Hermite polynomials. Thus, ${\left\{K_{n}\left(x\right)\right\}}_{n\in N}$ is an orthonormal basis in $L^{2}\left(R\right)$. As is well known,

(9) $$\int_{-\infty }^{\infty }K_{n}\left(x\right)\;K_{m}\left(x\right)\;dx=\delta_{nm},\;\sum_{n=0}^{\infty }K_{n}\left(x\right)\;K_{n}\left(x^{\prime }\right)=\delta \left(x-x^{\prime }\right).$$

Note that we denote by N the set of positive integers or natural numbers together with 0 and by $N^{*}=N-\left\{0\right\}$.

We see that the spectrum of the operator *N* is countably infinite, so that we may construct a countable orthonormal basis of eigenvectors of *N*, ${\{|n\rangle \}}_{n\in N}$, in terms of the continuous basis related to *X* and the Hermite functions. This is given by the following relation:

(10) $$|n\rangle :={\left(2\pi \right)}^{-1/4}\int_{-\infty }^{\infty }K_{n}\left(x\right)\;|x\rangle \;dx,\;n=0,1,2,\cdots .$$

From the properties of the continuous basis as well as of the Hermite functions, we obtain that

(11) $$\langle n|m\rangle =\delta_{nm},\;\sum_{n=0}^{\infty }\left|n\rangle \langle n\right|=I.$$

It is worth noticing that the Hermite functions are eigenfunctions of the Fourier transform:

(12) $$\left[F\;K_{n}\right]\left(p\right)\equiv {\tilde{K}}_{n}\left(p\right)=\frac{1}{\sqrt{2\pi }}\int_{-\infty }^{\infty }e^{ipx}\;K_{n}\left(x\right)\;dx=i^{n}\;K_{n}\left(p\right).$$

This expression allows us to write relations between the three bases: one discrete and two continuous, which have been defined in this section. These relations are

(13) $$\begin{array}{ccc} |n\rangle & = & i^{n}{\left(2\pi \right)}^{-1/4}\int_{-\infty }^{\infty }K_{n}\left(p\right)\;|p\rangle \;dp, \end{array}$$

(14) $$\begin{array}{ccc} |x\rangle & = & {\left(2\pi \right)}^{1/4}\sum_{n=0}^{\infty }K_{n}\left(x\right)\;|n\rangle , \end{array}$$

(15) $$\begin{array}{ccc} |p\rangle & = & {\left(2\pi \right)}^{1/4}\sum_{n=0}^{\infty }i^{n}\;K_{n}\left(p\right)\;|n\rangle \;. \end{array}$$

We see that the Hermite functions are the elements of the “transition matrices” between the continuous ad the discrete bases. We can express any ket |f〉 in any of the three bases in terms of the following equations:

(16) $$|f\rangle =\frac{1}{\sqrt{2\pi }}\int_{-\infty }^{\infty }dx\;f\left(x\right)\;|x\rangle \;,\;|f\rangle =\frac{1}{\sqrt{2\pi }}\int_{-\infty }^{\infty }dp\;\tilde{f}{\left(p\right)}^{*}\;|p\rangle \;,\;|f\rangle ={\left(2\pi \right)}^{-1/4}\;\sum_{n=0}^{\infty }a_{n}\;|n\rangle ,$$

with

(17) $$f\left(x\right):=\langle x|f\rangle =\sum_{n=0}^{\infty }\;c_{n}\;K_{n}\left(x\right),\;\tilde{f}{\left(p\right)}^{*}:=\langle p|f\rangle =\sum_{n=0}^{\infty }{\left(-i\right)}^{n}\;c_{n}\;K_{n}\left(p\right),$$

and

(18) $$c_{n}={\left(2\pi \right)}^{1/4}\;\langle n|f\rangle =\int_{-\infty }^{\infty }dx\;K_{n}\left(x\right)\;f\left(x\right)=i^{n}\;{\left(2\pi \right)}^{-1/4}\int_{-\infty }^{\infty }dp\;K_{n}\left(p\right)\;\tilde{f}{\left(p\right)}^{*}.$$

Therefore, we have obtained three different manners of expressing a quantum state |f〉 in terms of three different bases: two of them are continuous and non-countable, ${\{|x\rangle \}}_{x\in R}$ and ${\{|p\rangle \}}_{p\in R}$, and the other one, ${\{|n\rangle \}}_{n\in N}$, is countably infinite. The framework to deal together with all three of these bases is the RHS [21].

In particular, the set $\{|n\rangle \equiv K_{n}{\left(x\right)\}}_{n\in N}$ is a discrete basis of Φ≡S (the Schwartz space) and $H\equiv L^{2}\left(R\right)$ and the continuous bases belong to $\Phi^{\times }\equiv S^{\times }$ (the space of tempered distributions). More precisely, we have two equivalent RHS: one is abstract $\Phi \subset H\subset \Phi^{\times }$ and the other admits a realization in terms of functions, $S\subset L^{2}\left(R\right)\subset S^{\times }$. They are related through the unitary map $U:H⟼L^{2}\left(R\right)$ defined by $U|n\rangle =K_{n}\left(x\right)$. There is another interesting fact related with the use of RHS: the space S belongs to the domain of the operators in UEA[io(2)]. All of these operators can be extended by duality to continuous (under any topology on $S^{\times }$ compatible with the dual pair) operators on $S^{\times }$. For a detailed exposition of the actual case, see [6] and references therein.

### 2.2. UEA[io(2)] and Fractional Fourier Transform

Let us consider the kets |n〉 that form a complete orthonormal system in the abstract Hilbert space H. For any n∈N and 0<k≤n∈N, we consider the natural numbers, *q* and *r* such that n=kq+r, where r=0,1,2,⋯,k−1. For *k* fixed, the set {|kq+r〉} is a complete orthonormal system in H. Let us define the operators *Q* and *R* as

(19) $$\begin{array}{c} Q\;|k\;q+r\rangle :=q\;|k\;q+r\rangle ,\;R\;|k\;q+r\rangle :=r\;|k\;q+r\rangle \;. \end{array}$$

These operators also act on Φ⊂H and can be extended by duality to $\Phi^{\times }$.

Infinitely many copies of the Lie algebra io(2) are contained in UEA[io(2)]. Thus, for any positive integer *k*, each of the pairs (k,r) with 0≤r≤k−1 labels a copy of io(2), here denoted as $io_{k,r}\left(2\right)$. Furthermore,

$$\overset{k-1}{\underset{r=0}{⨁}}\;io_{k,r}\left(2\right)\subset UEA\left[io\left(2\right)\right].$$

We define the family of operators $A_{k,r}^{†}$ and $A_{k,r}$ in UEA[io(2)] by

$$A_{k,r}^{†}:={\left(a^{†}\right)}^{k}\frac{\sqrt{N+k-r}}{\sqrt{k\prod_{j=1}^{k}\left(N+j\right)}},\;A_{k,r}:=\frac{\sqrt{N+k-r}}{\sqrt{k\prod_{j=1}^{k}\left(N+j\right)}}{\left(a\right)}^{k},$$

where $A_{k,r}^{†}$ is the formal adjoint of $A_{k,r}$ and viceversa. They are continuous on Φ and can be continuously extended by duality to $\Phi^{\times }$. Their action on the vectors |kq+r〉 is

$$A_{k,r}^{†}\;|k\;q+r\rangle =\sqrt{q+1}\;|k\;\left(q+1\right)+r\rangle \;,\;A_{k,r}\;|k\;q+r\rangle =\sqrt{q}\;|k\;\left(q-1\right)+r\rangle .$$

For each pair of integers *k* and *r* with 0≤r<k, the operators $Q,A_{k,r}^{†},A_{k,r}$ and I close a io(2) Lie algebra, here denoted as $io_{k,r}\left(2\right)$. The commutation relations are

$$\begin{array}{cccccc} \left[Q,\;A_{k,r}^{†}\right] & = & +\;A_{k,r}^{†},\; & \left[Q,A_{k,r}\right] & = & -\;A_{k,r},\\ \left[A_{k,r},\;A_{k,r}^{†}\right] & = & I,\; & \left[I,\cdot \right] & = & 0\;. \end{array}$$

Note that, for any pair (k,r), the kets |kq+r〉 span subspaces $H_{k,r}$ of H and $L_{k,r}^{2}\left(R\right)$ of $L^{2}\left(R\right)$. Hence, we have that

(20) $$H=\overset{k-1}{\underset{r=0}{⨁}}H_{k,r},\;L^{2}\left(R\right)=\overset{k-1}{\underset{r=0}{⨁}}L_{k,r}^{2}\left(R\right).$$

We can easily obtain the spaces $\Phi_{k,r}$ and $S_{k,r}$. A vector |ϕ〉 belongs to $\Phi_{k,r}$ if and only if

(21) $$|\phi \rangle =\sum_{q=0}^{\infty }c_{q}\;|k\;q+r\rangle ,$$

such that

$$\sum_{q=0}^{\infty }{\left(q+1\right)}^{2p}\;{\left|c_{q}\right|}^{2}\;<\infty ,\;\forall p\in N.$$

A similar result can be obtained for any $S_{k,r}$, just replacing |kq+r〉 by $K_{k\;q+r}\left(x\right)$ in Label (21). Moreover, the corresponding RHS can be obtained:

(22) $$\begin{array}{c} \Phi_{k,r}\subset H_{k,r}\subset \Phi_{k,r}^{\times },\\ S_{k,r}\subset L_{k,r}^{2}\left(R\right)\subset S_{k,r}^{\times }\;. \end{array}$$

One can also prove that an operator O belongs to $UEA[io_{k,r}\left(2\right)]$ if and only if O is an operator on $H_{k,r}$.

The split of $L^{2}\left(R\right)$ as a direct sum of subspaces $L_{k,r}^{2}\left(R\right)$ is connected with the FFT, which is is a generalization of the Fourier transform [36]. It is very interesting that we can also relate the FFT with the Hermite functions $K_{n}\left(x\right)$ (8) in a simple manner. Let us first define the *fractional Fourier transform* of $f\in L^{2}\left(R\right)$ associated to a∈R, $F^{a}f$, as

(23) $$\left[F^{a}f\right]\left(p\right):=\sum_{n=0}^{\infty }c_{n}\;e^{i\;n\;a\;\pi /2}\;K_{n}\left(p\right),$$

where

(24) $$f\left(x\right)=\sum_{n=0}^{\infty }c_{n}\;K_{n}\left(x\right),\;c_{n}=\int_{-\infty }^{\infty }f^{*}\left(x\right)\;K_{n}\left(x\right)\;dx.$$

The convergence of the series in (23) is in the $L^{2}\left(R\right)$ norm as well as in the more generalized sense given in (21) if f(x)∈S, so that $F^{a}f\in S$ if f∈S.

When a=4/k, with $k\in N^{*}$, we have

(25) $${\tilde{f}}^{k}\left(p\right):=\left[F^{4/k}\;f\right]\left(p\right)=\sum_{n=0}^{\infty }c_{n}\;e^{2\;\pi \;i\;n/k}\;K_{n}\left(p\right).$$

In this case, we recover the standard Fourier transform for k=4, which means that a=1. Since for every $k\in N^{*}$, any n∈N can be decomposed as n=kq+r with q∈N and 0≤r≤k−1, we have the following decomposition of ${\tilde{f}}^{k}$ given by

(26) $$\begin{array}{ccc} {\tilde{f}}^{k}\left(p\right) & = & \sum_{q=0}^{\infty }c_{kq}\;e^{2\pi (kq)i/k}\;K_{kq}\left(p\right)+\sum_{q=0}^{\infty }c_{kq+1}\;e^{2\pi (kq+1)i/k}\;K_{kq+1}\left(p\right)\\ & & \;+\cdots +\sum_{q=0}^{\infty }c_{kq+k-1}\;e^{2\pi (kq+k-1)i/k}\;K_{kq+k-1}\left(p\right)\\ & = & {\tilde{f}}_{0}^{k}\left(p\right)+{\tilde{f}}_{1}^{k}\left(p\right)+\cdots +{\tilde{f}}_{k-1}^{k}\left(p\right), \end{array}$$

where

$$f_{r}^{k}\left(x\right):=\sum_{q=0}^{\infty }c_{kq+r}\;K_{kq+r}\left(x\right),\;{\tilde{f}}_{r}^{k}\left(p\right):=e^{2\;\pi \;r\;i/k}\;f_{r}^{k}\left(p\right).$$

Relation (26) gives a split of $L^{2}\left(R\right)$ into an orthonormal direct sum of subspaces because the vectors ${\tilde{f}}_{r}^{k}$, (r=0,1,⋯,k−1) are mutually orthogonal. Moreover, each term in the direct sum is an eigen-subspace of $F^{4/k}$ with eigenvalue $e^{i2\pi r/k}$ since ${\tilde{f}}_{r}^{k}\left(p\right)\equiv \left[F^{4/k}\;f_{r}^{k}\right]\left(p\right)$. The decomposition is given by

$$L^{2}\left(R\right)=L_{k,0}^{2}\left(R\right)⊕L_{k,1}^{2}\left(R\right)⊕\cdots ⊕L_{k,k-1}^{2}\left(R\right),$$

so that we have recovered the decomposition (20).

## 3. Harmonic Analysis on $R^{+}$

Let $L^{2}\left(R^{+}\right)$ be the space of square integrable functions on $R^{+}\equiv \left[0,+\infty \right)$. As is well known a basis in $L^{2}\left(R^{+}\right)$ is determined by the Laguerre functions

(27) $$M_{n}^{\alpha }\left(y\right)=\sqrt{\frac{\Gamma (n+1)}{\Gamma (n+\alpha +1)}}\;y^{\alpha /2}\;e^{-y/2}\;L_{n}^{\alpha }\left(y\right),$$

with −1<α<+∞, n=0,1,2,⋯, and $L_{n}^{\alpha }\left(y\right)$ the associated Laguerre polynomials [41,42]. Indeed, $M_{n}^{\alpha }\left(y\right)$ verify the following orthonormality and completeness relations

(28) $$\int_{0}^{\infty }M_{n}^{\alpha }\left(y\right)\;\;M_{m}^{\alpha }\left(y\right)\;dy\;=\;\delta_{nm},\;\sum_{n=0}^{\infty }\;M_{n}^{\alpha }\left(y\right)\;\;M_{n}^{\alpha }\left(y^{\prime }\right)\;=\;\delta \left(y-y^{\prime }\right).$$

### 3.1. Harmonic Analysis on su(1,1)

Let us define the following operators on $L^{2}\left(R^{+}\right)$

(29) $$\begin{array}{cccccc} N\;M_{n}^{\alpha }\left(y\right) & := & n\;M_{n}^{\alpha }\left(y\right), & I\;M_{n}^{\alpha }\left(y\right) & := & M_{n}^{\alpha }\left(y\right),\\ Y\;M_{n}^{\alpha }\left(y\right) & := & y\;M_{n}^{\alpha }\left(y\right), & D_{y}\;M_{n}^{\alpha }\left(y\right) & := & M_{n}^{\alpha }{\left(y\right)}^{\prime }=\frac{d}{dy}M_{n}^{\alpha }\left(y\right)\;. \end{array}$$

Using the operators defined in (29), we may define some others:

(30) $$J_{+}:=\left(Y\;D_{y}+N+1+\frac{\alpha -Y}{2}\;\right),\;J_{-}:=\left(-Y\;D_{y}+N+\frac{\alpha -Y}{2}\right).$$

These operators act on $M_{n}^{\alpha }\left(y\right)$ as

(31) $$\begin{array}{c} J_{+}M_{n}^{\alpha }\left(y\right)=\sqrt{\left(n+1)(n+\alpha +1\right)}M_{n+1}^{\alpha }\left(y\right),\\ J_{-}M_{n}^{\alpha }\left(y\right)=\sqrt{n(n+\alpha )}M_{n-1}^{\alpha }\left(y\right). \end{array}$$

These two operators, together with

$$J_{3}:=N+\frac{\alpha +1}{2}\;I,\;J_{3}\;M_{n}^{\alpha }\left(y\right)=\left(n+\frac{\alpha +1}{2}\right)\;M_{n}^{\alpha }\left(y\right),$$

define the Lie algebra su(1,1) because their commutation relations are

(32) $$\left[J_{3},J_{\pm }\right]=\pm J_{\pm },\;\left[J_{+},J_{-}\right]=-2\;J_{3}.$$

The Casimir operator C of su(1,1) is

(33) $$C=J_{3}^{2}-\frac{1}{2}\left\{J_{+},J_{-}\right\}=\frac{\alpha^{2}-1}{4}\;I.$$

From (30), we obtain that $Y=-\left(J_{+}+J_{-}\right)+2N+\left(\alpha +1\right)\;I,$ and from the Casimir we may obtain the differential equation defining the associated Laguerre polynomials.

Omitting the technical details which can be found in Reference [6], let us say that there exists a set of generalized eigenvectors of *Y*, ${\{|y\rangle \}}_{y\in R^{+}}$, (or more strictly of $U^{-1}YU$, where $U:H⟼L^{2}\left(R^{+}\right)$ is a unitary operator and H is a separable Hilbert space) such that

(34) $$Y|y\rangle =y|y\rangle \;,\;\;\langle y|y^{\prime }\rangle =\delta \left(y-y^{\prime }\right),\;\int_{-\infty }^{+\infty }|y\rangle \langle y|\;dy=I.$$

Actually, we have two families, depending on α, of equivalent RHS $\Phi_{\alpha }\subset H\subset \Phi_{\alpha }^{\times }$ and $D_{\alpha }\subset L^{2}\left(R^{+}\right)\subset D_{\alpha }^{\times }$. All the elements and their extensions of the UEA(su(1,1)) are continuous on both RHS.

In analogy with the case of the whole real line, a decomposition like (20) for any k≠0∈N, we also obtain here that

(35) $$H=\overset{k-1}{\underset{r=0}{⨁}}H_{k,r},\;L^{2}\left(R^{+}\right)=\overset{k-1}{\underset{r=0}{⨁}}L_{k,r}^{2}\left(R^{+}\right).$$

We define the vectors $|n,\alpha \rangle \in \Phi_{\alpha }$ as

(36) $$|n,\alpha \rangle :=\int_{0}^{\infty }dy\;M_{n}^{\alpha }\left(y\right)|y\rangle ,\;\forall n\in N,\;\;\alpha \in \left(-1,+\infty \right),$$

which after (28) and (34), they have the properties

(37) $$\langle n,\alpha |m,\alpha \rangle =\delta_{nm},\;\sum_{n=0}^{\infty }\left|n,\alpha \rangle \langle n,\alpha \right|=I.$$

Hence, |n,α〉 with n∈N (and α fix) is an orthonormal basis in H. Taking into account the unitarity of the operator *U*, we have that $U|n,\alpha \rangle =M_{n}^{\alpha }$. For y≥0, we easily obtain

(38) $$\langle y|n,\alpha \rangle =\int_{0}^{\infty }dy^{\prime }\;M_{n}^{\alpha }\left(y^{\prime }\right)\;\langle y|y^{\prime }\rangle =M_{n}^{\alpha }\left(y\right).$$

In analogy with the previous case in which we have considered functions on the whole real line, we also have two different bases spanning the vectors in $\Phi_{\alpha }\subset H$: a continuous one ${\{|y\rangle \}}_{y\in R^{+}}$ and another discrete ${\{|n,\alpha \rangle \}}_{n\in N}$ whose elements are eigenvectors of the operator $U^{-1}NU$ where *N* was defined in (29).

### 3.2. Fourier-Like Transformations on $R^{+}$

In Section 2.2, we have introduced the FFT related to the Hermite functions. Now, after the results displayed in the previous section that show a close analogy between the formalisms on R and on $R^{+}$, we may consider an extension of the FFT valid for the generalized Laguerre functions. However, this is not possible since the Laguerre functions, $M_{n}^{\alpha }\left(y\right)$, are not eigenfunctions of the Fourier transform unlike the Hermite functions. Fortunately, there exists a partial way out due to the relations among Hermite and Laguerre polynomials

$$H_{2n}\left(x\right)={\left(-1\right)}^{n}\;2^{2n}\;n!\;L_{n}^{-1/2}\left(x^{2}\right),\;H_{2n+1}\left(x\right)={\left(-1\right)}^{n}\;2^{2n+1}\;n!\;x\;L_{n}^{+1/2}\left(x^{2}\right)\;$$

that allow us to relate the above-mentioned functions as

$$K_{2n}\left(x\right)={\left(-1\right)}^{n}\;{\left(x^{2}\right)}^{1/4}\;\;M_{n}^{-1/2}\left(x^{2}\right),\;K_{2n+1}\left(x\right)={\left(-1\right)}^{n}\;x\;{\left(x^{2}\right)}^{-1/4}\;\;M_{n}^{1/2}\left(x^{2}\right).$$

Thus, we can define the transforms $T_{\pm }$ on functions $f\left(y\right)\in L^{2}\left(R^{+}\right)$ by

(39) $$\left[T_{\pm }f\right]\left(s\right):=\frac{1}{\sqrt{2\pi }}\int_{0}^{\infty }dy\;\frac{S_{\pm }\left(\sqrt{s\;y}\right)}{{\left(s\;y\right)}^{1/4}}\;f\left(y\right),\;S_{+}\left(\cdot \right)=\cos\left(\cdot \right),\;\;S_{-}\left(\cdot \right)=\sin\left(\cdot \right),$$

such that they verify the relation

(40) $$\left[T_{\pm }M_{n}^{\pm 1/2}\right]\left(s\right)={\left(-1\right)}^{n}\;M_{n}^{\pm 1/2}\left(s\right),$$

which means that $M_{n}^{\pm 1/2}\left(s\right)$ are eigenfunctions with eigenvalues ${\left(-1\right)}^{n}$ of $T_{\pm }$. In consequence, we have two relevant values of the label α: ±1/2. Then, since, for any $f\left(y\right)\in L^{2}\left(R^{+}\right)$,

(41) $$f\left(y\right)=\sum_{n=0}^{\infty }c_{n}^{\pm }\;M_{n}^{\pm 1/2}\left(y\right),\;c_{n}^{\pm }=\int_{0}^{\infty }f^{*}\left(y\right)M_{n}^{\pm 1/2}\left(y\right)\;dy.$$

We may introduce two new families of FFT $T_{\pm }^{a}$ (a∈R) by

$$\left[T_{\pm }^{a}\;f\right]\left(s\right):=\sum_{n=0}^{\infty }c_{n}^{\pm }\;e^{i\;n\;a\;\pi /2}\;M_{n}^{\pm 1/2}\left(s\right).$$

Thus, if we choose a=4/k with $k\in N^{*}$, we have

(42) $$\begin{array}{ccc} {\tilde{f}}_{\pm }^{k}\left(s\right) & := & \left[T_{\pm }^{4/k}f\right]\left(s\right)=\sum_{q=0}^{\infty }c_{kq}^{\pm }\;e^{-2\pi (kq)i/k}\;M_{kq}^{\pm 1/2}\left(s\right)+\sum_{q=0}^{\infty }c_{kq+1}^{\pm }\;e^{-2\pi (kq+1)i/k}\;M_{kq+1}^{\pm 1/2}\left(s\right)+\cdots \\ & & \;\;\;\cdots +\sum_{q=0}^{\infty }c_{kq+k-1}^{\pm }\;e^{-2\pi (kq+k-1)i/k}\;M_{kq+k-1}^{\pm 1/2}\left(s\right)\\ & = & f_{0,\pm }^{k}\left(s\right)+e^{-2\pi i/k}\;f_{1,\pm }^{k}\left(s\right)+\cdots +e^{-2\pi (k-1)i/k}\;f_{k-1,\pm }^{k}\left(s\right), \end{array}$$

with

$$f_{r,\pm }^{k}\left(s\right):=\sum_{q=0}^{\infty }c_{kq+r}^{\pm }\;M_{kq+r}^{\pm 1/2}\left(s\right).$$

We have recovered the splitting (35) of $L^{2}\left(R^{+}\right)$ for the particular cases of α=±1/2

$$\begin{array}{c} L^{2}\left(R^{+}\right)=L_{k,0}^{2}{\left(R^{+}\right)}^{\pm }⊕L_{k,1}^{2}{\left(R^{+}\right)}^{\pm }⊕\cdots ⊕L_{k,k-1}^{2}{\left(R^{+}\right)}^{\pm }, \end{array}$$

where each of the closed subspaces $L_{k,r}^{2}{\left(R^{+}\right)}^{\pm }$ is an eigen-subspace of $T_{\pm }$ with eigenvalue $e^{-2\;\pi \;r\;i/k}$.

## 4. A New Harmonic Analysis on the Circle

The set Hermite functions $K_{n}\left(x\right)$ is a good tool so as to construct a countable set of periodic functions, which is a system of generators of the space of square integrable functions on the unit circle $L^{2}\left(C\right)$, i.e., the functions f(ϕ):C⟼C with norm ||f(ϕ)|| defined by

(43) $${\left||f\left(\phi \right)|\right|}^{2}:=\frac{1}{2\pi }\;\int_{-\pi }^{\pi }{\left|f\left(\phi \right)\right|}^{2}\;d\theta <\infty .$$

Let us define the periodic functions (with period 2π)

(44) $$K_{n}\left(\phi \right):=\sum_{k=-\infty }^{\infty }K_{n}\left(\phi +2k\pi \right),\;-\pi \leq \phi <\pi ,\;\;n=0,1,2,\cdots .$$

It can be proven that the series defining the $K_{n}\left(\phi \right)$ are absolutely convergent and also that every $K_{n}\left(\phi \right)$ is bounded and square integrable on the interval −π≤ϕ<π. Using this property and the Lebesgue theorem, we may also prove that

(45) $$\begin{array}{c} \int_{-\pi }^{\pi }e^{im\phi }\;d\phi \sum_{k=-\infty }^{\infty }K_{n}\left(\phi +2k\pi \right)=\sum_{k=-\infty }^{\infty }\int_{-\pi }^{\pi }e^{im\phi }\;K_{n}\left(\phi +2k\pi \right)\;d\phi \;. \end{array}$$

### A Discretized Fourier Transform on the Circle

Let us compare the space $L^{2}\left(C\right)$, which we may also denote as $L^{2}\left[-\pi ,\pi \right]$, to the space $l_{2}\left(Z\right)$ of 2-power summable sequences. As is well known, an orthonormal basis on $L^{2}\left(C\right)$ is $\left\{{\left(2\pi \right)}^{-1}\;e^{in\phi }\right\}$ with n∈Z. Hence, any $f\left(\phi \right)\in L^{2}\left(C\right)$ admits the following span into exponential Fourier series given by

(46) $$f\left(\phi \right)=\frac{1}{2\pi }\;\sum_{n\in Z}f_{n}\;e^{in\phi },\;f_{n}\in C,$$

with

(47) $$f_{n}=\frac{1}{2\pi }\;\int_{-\pi }^{\pi }f\left(\phi \right)\;e^{-in\phi }\;d\phi .$$

The sum (46) converges in the sense of the norm (43). Moreover, for any continuous function f(ϕ), the series also converge pointwise. The properties of orthonormal basis in Hilbert spaces show that

(48) $$\frac{1}{2\pi }\;\sum_{n\in Z}|f_{n}{|}^{2}={\left\|f\left(\phi \right)\right\|}^{2}.$$

We may call to the sequence of complex numbers ${\left\{f_{n}\right\}}_{n\in Z}$, the components of *f*.

The Hilbert space $l_{2}\left(Z\right)$ is a space of sequences of complex numbers $A\equiv {\left\{a_{n}\right\}}_{n\in Z}$ such that

(49) $${\left\|A\right\|}^{2}:=\frac{1}{2\pi }\;\sum_{n\in Z}{\left|a_{n}\right|}^{2}<\infty ,$$

with scalar product given by

(50) $$\langle A|B\rangle :=\frac{1}{2\pi }\sum_{n\in Z}a_{n}^{*}\;b_{n}.$$

An orthonormal basis for $l_{2}\left(Z\right)$ is given by the sequences $E_{k}={\left\{\delta_{k,n}\right\}}_{n\in Z}$ with k∈Z. Any $f\in l_{2}\left(Z\right)$ with components ${\left\{f_{n}\right\}}_{n\in Z}$ may be written as

(51) $$f=\frac{1}{2\pi }\sum_{n\in Z}f_{n}\;E_{n},\;\frac{1}{2\pi }\;\sum_{n\in Z}|f_{n}{|}^{2}={\left\|f\right\|}^{2}<+\infty .$$

Therefore, there exists a unitary correspondence between $L^{2}\left(C\right)$ and $l_{2}\left(Z\right)$ which maps any $f\left(\phi \right)\in L^{2}\left(C\right)$ as in (46) into *f* as in (51), provided that in both cases the sequence ${\left\{f_{n}\right\}}_{n\in Z}$ is the same.

Expression (46) gives the expansion into Fourier series of the functions in $L^{2}\left(C\right)$. From this point of view, we may say that the Fourier series is a unitary mapping, F, from $L^{2}\left(C\right)$ onto $l_{2}\left(Z\right)$. It admits an inverse, $F^{-1}$, from $l_{2}\left(Z\right)$ onto $L^{2}\left(C\right)$, which is also unitary and is sometimes called the discrete Fourier transform, i.e.,

(52) $$F\left[f\left(\phi \right)\right]=\frac{1}{2\pi }\;\sum_{n\in Z}f_{n}\;e^{in\phi }\equiv {\left\{f_{n}\right\}}_{n\in N},\;F^{-1}\left[{\left\{a_{n}\right\}}_{n\in N}\right]=\frac{1}{2\pi }\;\sum_{n\in Z}a_{n}\;e^{in\phi }\equiv a\left(\phi \right),$$

with $f_{n}\in C$ and given by (47).

As we mention in the introduction, we will give a unitary version of concepts that are often introduced separately, like Fourier transform, Fourier series and discrete Fourier transform in one side and the Hermite functions on the other.

We start by constructing a set of functions in $l_{2}\left(Z\right)$ using the Hermite functions $K_{n}\left(x\right)$. We introduce the sequences $\chi_{n}$ associated to $K_{n}\left(x\right)$ as follows:

(53) $$\chi_{n}:={\left\{K_{n}\left(m\right)\right\}}_{m\in Z},\;n\in N.$$

These sequences $\chi_{n}$ are in $l_{2}\left(Z\right)$. Moreover, they are linearly independent and span $l_{2}\left(Z\right)$. The proof can be find in [43] and they are based on the fact that

(54) $$\left|\begin{array}{ccccc} H_{0}\left(-N\right) & \cdots & H_{0}\left(0\right) & \cdots & H_{0}\left(N\right)\\ H_{1}\left(-N\right) & \cdots & H_{1}\left(0\right) & \cdots & H_{1}\left(N\right)\\ \cdots & \cdots & \cdots & \cdots & \cdots \\ H_{2N}\left(-N\right) & \cdots & H_{2N}\left(0\right) & \cdots & H_{2N}\left(N\right) \end{array}\right|\neq 0,$$

and

(55) $$\left|\begin{array}{cccc} H_{0}\left(0\right) & H_{0}\left(1\right) & \cdots & H_{0}\left(N\right)\\ H_{1}\left(0\right) & H_{1}\left(1\right) & \cdots & H_{1}\left(N\right)\\ \cdots & \cdots & \cdots & \cdots \\ H_{N}\left(0\right) & H_{N}\left(1\right) & \cdots & H_{N}\left(N\right) \end{array}\right|\neq 0,$$

for any N∈N, where $H_{n}\left(k\right)$ is the Hermite polynomial $H_{n}\left(x\right)$ evaluated at the integer *k* (remember that $K_{n}\left(x\right)=e^{-x^{2}/2}\;H_{n}\left(x\right)/\sqrt{2^{n}n!\sqrt{\pi }}$).

Since the functions $K_{n}\left(\phi \right)$ are in $L^{2}\left[-\pi ,\pi \right]$, they admit a span in terms of the orthonormal basis ${\left\{{\left(2\pi \right)}^{-1}\;e^{im\phi }\right\}}_{m\in Z}$ in $L^{2}\left[-\pi ,\pi \right]$. Thus, we can write

(56) $$K_{n}\left(\phi \right)=\frac{1}{\sqrt{2\pi }}\sum_{m=-\infty }^{\infty }k_{n}^{m}\;e^{-im\phi },$$

with

(57) $$k_{n}^{m}=\frac{1}{\sqrt{2\pi }}\int_{-\pi }^{\pi }e^{im\phi }\;K_{n}\left(\phi \right)\;d\phi .$$

The continuity of the functions $K_{n}\left(\phi \right)$ on [−π,π] guarantees the pointwise convergence of (56) and since the $K_{n}\left(\phi \right)$ are periodic with period 2π, hence (56) is valid for all ϕ∈R.

We recall that the Hermite functions $K_{n}\left(x\right)$ are eigenfunctions of the Fourier transform with eigenvalue ${\left(-i\right)}^{n}$ (12), i.e., $\left[F\;K_{n}\right]\left(p\right)={\left(-i\right)}^{n}\;K_{n}\left(p\right)$. Thus, $K_{n}\left(x\right)$ are eigenfunctions of the inverse Fourier transform with eigenvalue $i^{n}$, i.e., $\left[F^{-1}\;K_{n}\right]\left(x\right)=i^{n}\;K_{n}\left(x\right)$. From this fact, we can find an explicit expression of the coefficients $k_{n}^{m}$ (57) in terms of the values of the Hermite functions at the integers

$$\begin{array}{ccc} k_{n}^{m} & = & \frac{1}{\sqrt{2\pi }}\int_{-\pi }^{\pi }e^{im\phi }\;d\phi \left[\sum_{k=-\infty }^{\infty }K_{n}\left(\phi +2k\pi \right)\right]=\frac{1}{\sqrt{2\pi }}\sum_{k=-\infty }^{\infty }\int_{-\pi }^{\pi }e^{im\phi }\;K_{n}\left(\phi +2k\pi \right)\;d\phi \\ & = & \frac{1}{\sqrt{2\pi }}\sum_{k=-\infty }^{\infty }\int_{-\pi +2k\pi }^{\pi +2k\pi }e^{ims}\;K_{n}\left(s\right)\;ds=\frac{1}{\sqrt{2\pi }}\int_{-\infty }^{\infty }e^{ims}\;K_{n}\left(s\right)\;ds=i^{n}\;K_{n}\left(m\right)\;, \end{array}$$

where s:=ϕ+2kπ and $e^{im\phi }=e^{im(\phi +2k\pi )}=e^{ims}$. Hence, (56) and (57) can be written, respectively, as

(58) $$K_{n}\left(\phi \right)=\frac{i^{n}}{\sqrt{2\pi }}\sum_{m=-\infty }^{\infty }K_{n}\left(m\right)\;e^{-im\phi },\;K_{n}\left(m\right)=\frac{{\left(-i\right)}^{n}}{\sqrt{2\pi }}\int_{-\pi }^{\pi }K_{n}\left(\phi \right)\;e^{im\phi }\;d\phi ,$$

where $k_{n}^{m}=i^{n}\;K_{n}\left(m\right)$.

The systems of generators in $L^{2}\left[-\pi ,\pi \right]\equiv L^{2}\left(C\right)$, ${\left\{K_{n}\left(\phi \right)\right\}}_{n\in Z}$, and in $l_{2}\left(Z\right)$, given by the set of sequences ${\left\{\chi_{n}\right\}}_{n\in Z}$, are not orthonormal basis. The scalar product on $L^{2}\left[-\pi ,\pi \right]$ is related with the scalar product in $l_{2}\left(Z\right)$

$$\begin{array}{cc} \langle K_{n}|K_{m}\rangle & =\int_{-\pi }^{\pi }K_{n}^{*}\left(\phi \right)\;K_{m}\left(\phi \right)\;d\phi =\frac{1}{2\pi }\int_{-\pi }^{\pi }d\phi \sum_{j=-\infty }^{\infty }\sum_{k=-\infty }^{\infty }{\left(-i\right)}^{n}\;i^{m}\;K_{n}^{*}\left(j\right)\;K_{m}\left(k\right)\;e^{-i(k-j)\phi }\\ & =\sum_{j=-\infty }^{\infty }\sum_{k=-\infty }^{\infty }\delta_{j,k}\;i^{m-n}\;K_{n}^{*}\left(j\right)\;K_{m}\left(k\right)=\sum_{j=-\infty }^{\infty }i^{m-n}\;K_{n}^{*}\left(j\right)\;K_{m}\left(j\right)\\ & =i^{m-n}\;\left(\chi_{n},\chi_{m}\right)\;. \end{array}$$

The Gramm–Schmidt procedure allows us to obtain orthogonal bases in both spaces.

## 5. Conclusions

In this paper, we have presented a unified framework where Hermite functions, or alternatively Laguerre functions, their symmetry groups, Fourier analysis and RHS fit in a perfect manner. Hermite functions are basic in the study of quantum mechanics and signal processing on the real line R, while Laguerre functions play the same role on the half-line $R^{+}$. We have also studied the particular relation between both situations. In both cases, these functions are eigenvectors of the Fourier transform and this is an essential property.

It is precisely the use of RHS that allows the use of bases of different cardinality on a simple and interchangeable manner. This makes RHS the correct mathematical formulation that encompasses both quantum mechanics and signal processing. Here, Hermite functions act as transition elements of transition matrices between continuous and discrete bases. This is not strictly new as was already discussed in [21], although we introduce a general point of view which could be relevant for computational and epistemological purposes in quantum theory.

We have shown how Fourier analysis allows for the decomposition of RHS into direct sums of RHS. This may permit the filtering of noise or any other undesirable signal. The same applies to operators as we may restrict their evolution to a sub-algebra, which has been chosen among infinite other possibilities in the universal enveloping algebra of the corresponding symmetry group. The decomposition of RHS is consistent with the FFT. This is the cornerstone of the filtering procedure. We have extended the formalism to functions over the semi-axis $R^{+}$ by the construction of a pair of “Fourier-like” transformations which play the role before reserved to the Fourier transform on R. FFTs may be defined after these Fourier-like transforms and also serve for filtering. Moreover, the algebraic approach associated to the Lie symmetry algebra and its universal enveloping algebra extends the discussion from the vector spaces to the space of operators acting on them.

All of these results can be also translated, in some sense, to the circle. We have constructed some special functions on the circle out of Hermite functions and have taken advantage of the properties of Hermite functions in order to use Fourier analysis on the circle as well. This work is still in process.

As a final remark, let us insist that we have given a unitary point of view of mathematical objects that are often considered as unrelated such as Fourier transform, discrete Fourier transform, Hermite and Laguerre functions and RHS. 
